# SoC FPGA Accelerated Sub-Optimized Binary Fully Convolutional Neural Network for Robotic Floor Region Segmentation

**DOI:** 10.3390/s20216133

**Published:** 2020-10-28

**Authors:** Chi-Chia Sun, Afaroj Ahamad, Pin-He Liu

**Affiliations:** 1 Digital System Design Lab, National Formosa University, Huwei 632, Taiwan; d0877103@gm.nfu.edu.tw (A.A.); 10765119@gm.nfu.edu.tw (P.-H.L.); 2 Smart Machine and Intelligent Manufacturing Research Center, National Formosa University, Huwei 632, Taiwan

**Keywords:** UGV, Binary Neural Network (BNN), Fully Convolutional Network (FCN), Taguchi Method (TM), SoC-FPGA, floor segmentation, motion control

## Abstract

In this article, a new Binary Fully Convolutional Neural Network (B-FCN) based on Taguchi method sub-optimization for the segmentation of robotic floor regions, which can precisely distinguish floor regions in complex indoor environments is proposed. This methodology is quite suitable for robot vision in an embedded platform and the segmentation accuracy is up to 84.80% on average. A total of 6000 training datasets were used to improve the accuracy and reach convergence. On the other hand, to reach real-time computation, a PYNQ FPGA platform with heterogeneous computing acceleration was used to accelerate the proposed B-FCN architecture. Overall, robots would benefit from better navigation and route planning in our approach. The FPGA synthesis of our binarization method indicates an efficient reduction in the BRAM size to 0.5–1% and also GOPS/W is sufficiently high. Notably, the proposed faster architecture is ideal for low power embedded devices that need to solve the shortest path problem, path searching, and motion planning.

## 1. Introduction

The effects of machine learning on our everyday life have been far-reaching. During the last few years, artificial intelligence and machine learning have shown that they are effective and have a tremendous amount of utility in solving real-world computationally-intensive problems. The detection and classification of an image are very often to understand what and where is it. Regional segmentation has garnered a deal of interest, especially in terms of designing an indoor robotic unmanned ground vehicle. To detection and map the floor, image processing and computer vision are required. Technology for indoor robots has been flourishing and researchers have found significant applications for floor detection by Unmanned Group Vehicles (UGVs) [1]. The UGV allows the robotic vehicle to identify the walkable range on the ground so that it can share its visual range and environmental data, which requires high-performance ground-range-detection calculation design scope plays a very important role in robotics control. Another example of the application of regional segmentation is the Advanced Driver Assistance System (ADAS) [2]. ADAS employs an algorithm of regional segmentation that assists the driver in terms of having rules for driving and warns to driver of any kind of obstacle in its way of driving so that vehicle accidents can be avoided. The automatic driver faces significant challenges in discerning vehicles and preparing the rules for driving. The most common method in the previous literature was to use parallax or depth information from a dual lens to detect other object [3]. The calculation for this was completed under the conditions of supply, etc. At present, existing neural networks and machine learning [4], image recognition processing, heterogeneous image fusion [5], depth information ground segmentation methods [6], etc., all require a high degree of computing power. Moreover, the increased-complexity of the calculations needed for the ground area segmentation method means that is not an acceptable design method in this case.

The vehicle discernment results for accident safeguards include data for, maintaining a safe distance at the speed limit, and automatic headlamp dimming. The logistical regression and support vector machines (SVMs) [7] are a better option for simple pattern recognition, regarding computer vision. They provide an adequate and acceptable amount of accuracy and are cost-effective in comparison of neural networks. However, since vehicles have different from there shapes, colors, etc., this increases the pattern’s complexity. For this reason, Deep Neural Network (DNN) has advantages over the traditional classification models.

Over the few years, Deep Neural Networks (DNNs) has become an active area of research in the field of machine learning. DNNs have provided outstanding results in the areas of computer vision, the recognition of speech, statistical machine translation, natural language processing, regression, and robotics. The DNNs first differentiate objects in to simple pattern and use this simple pattern to identify the objects. Deep Convolutional Neural Networks (CNNs) [8] are widely use in DNNs for data analysis. The data may be image, video or speech. Using large-scale datasets several studies have found that CNNs have a tremendous amount of accuracy in terms of object recognition, even more than humans. Since, Deep CNNs provide an incredible amount of accuracy. However, this work requires a large dataset, its computational complexity requires an immense amount of storage. This requirement of CNNs makes it unstable for real-time embedded applications when a significant factor is having a low amount of power. For this reason, GPU [9] is the only option for CNNs with large datasets.

For a single image detection, contemporary CNNs may be required to perform billions of floating-point operations, which increases the need for storage. For instance, AlexNet [10] requires 244 MB of parameters and 1.4 billion floating-point operations (GFLOPs) per image, while VGG-16 [11] needs 552 MB of parameters and 30.8 GFLOPs per image. Thus, we need to shorten the data storage and computational stage of DNNs. In this way, a 32-bit float point can be converted into low precision bits using quantization of a float point [12]. Quantization refers to the process of reducing the float number into a low precision number i.e., bits. A Binary Neural Network (BNN), a binary quantized version of CNNs, since it can significantly alleviate the SRAM/cache memory access overhead and on-chip storage constraints.

Low-precision arithmetic requires a smaller amount of memory for storage and less complexity. This advantages enhance the speed and make the operation more power-efficient in terms of image classification for Field Programmable Gate Arrays (FPGAs) Platform. For binary operations such as XOR, LUT, and Add, compare with floating-point operations FPGAs have a much higher theoretical peak performance. In addition, their small memory footprint removes the off-chip memory bottleneck by keeping the parameters on-chip, even when there are extensive networks. The BNN proposed by Courbariaux et al. 2015 [13], is interesting and can be implemented almost entirely with binary operations. It has the potential to attain a high performance in the tera-operations-per-second (TOPS) range on FPGAs.

Additionally, the FPGAs are more cost and power efficient than GPUs, and CPUs for embedded applications in machine learning. The significant factors associated with FPGAs are system performance and real-time applications. FPGAs are used as accelerators to compute, connect, collect, and process the massive quantities of information around us while employing a controlled data path. Moreover, the FPGAs have the ability of offload computing operation, i.e., they can directly receive data and process data inline without going through the host system. Hence, they stop the processor doing other work and provide higher real-time system performance. Therefore, by implementing binary-based machine learning on an FPGA platform, we can obtain a classification and segmentation system for the robot, that is efficient, reconfigurable, and has better power efficiency.

Dr. Taguchi of Nippon Telephones and Telegraph Company, Japan has proposed a statistical approach based on “ORTHOGONAL ARRAY” (Taguchi method) [14,15], which employs an experimental design for quality control to ensure excellent performance during the design stage of the process. Especially in the last few years, this method has been used in several analytic research studies [16,17,18,19] to design experiments with the best performance in fields like engineering, biotechnology, and computer science. Three factors should be consider when applying the Taguch method to find the best options i.e., the Taguchi loss function, offline quality control, and the orthogonal arrays for experimental design. Lin et al. [20] have suggested using Taguchi based CNNs that are optimized CNNs with an AlexNet Architecture. This CNN AlexNet architecture is used for gender image recognition. Lin et al. used the MORPH database and claimed to have obtained a 3.47% greater accuracy than the original AlexNet network. Another Taguchi based 2D CNN has been suggested [21]. They use Taguchi method to parametric optimization of 2D CNN to improve the computer tomography image in lung cancer recognition. They used two types of database, LIDC-IDRI and SPIE-AAPM, and claim 6.86% and 5.29% better performance than the original 2D CNN in the LIDC-IDRI and SPIE-AAPM databases, respectively. Another study [22] looked at the computer tomography image classification of the lung nodule. In this study, the authors suggested using a Taguchi based CNN architecture that improves the accuracy of the lung nodule classification of the computer tomography images obtaining an accuracy of 99.6%. In this paper, uses the Taguchi method to search for the sub-optimal B-FCN architecture that best balances hardware resource utilization and accuracy.

In this research, we focus on the segmentation of the image. Many neural network architectures have been previously suggested. Fully Convolutional Networks (FCNs) have a fundamental architecture proposed by Long et al. [23] for segmentation. Segmentation is more difficult task than classification or detection. It involves the labeling each pixel of the image. Semantic Segmentation Network (Seg-Net) [24,25] is a semantic analysis network model, whose structure is similar to the FCN. High-Resolution Networks (HR-Nets) [26] utilize a network architecture which is mainly for high-resolution data calculations and does not abandon high-resolution data sources to maintain high-resolution data characteristics. In multi-scale fusion of the network layer, each one is based on the branch feature images of different resolutions carried out on a collected sample. Here, we have chosen the FCN architecture for our segmentation architecture.

In this paper, the proposed floor segmentation method is based on the Binary Fully Convolutional Neural Network (B-FCN) using the FPGA accelerator. We search a sub-optimal B-FCN architecture using the Taguchi method. The BNN accelerator on an FPGA has an exceptionally good image classification speed, and accuracy. In addition, in the B-FCN, the operation and required memory size are smaller. The experimental results show that the proposed method accuracy is awfully close to the traditional Fully Convolutional Neural Network. The advantages of the present architecture are (1) the accuracy is upward of 87.58% on average compared with other methods reported in the literature; (2) the B-FCN accelerator on FPGA uses less memory storage BRAM size of the ZCU104 is only 0.5%−1% in terms of a fast image segmentation process; (3) it is worth noting that the proposed fast methodology is ideal for UGV rovers using embedded devices that need to solve path searching in motion planning for real-time operations with a high amount of energy efficiency.

Onward, this paper is organized as follows. In Section 2 we summarize the Quantization Neural Networks algorithm, as it concerns deep learning. Section 3 reviews the different Binary Neural Networks Model. Section 4 presents the proposed sub-optimal method of FPGA hardware accelerator based on Binary Fully Convolutional Neural Network architecture using Taguchi method. The experimental results and hardware implementation are reviewed in Section 5. Lastly, Section 6 concludes the research.

## 2. Quantization Neural Network

In Section 2, we describe the quantization of CNNs. Quantization [27] allows reducing the float numbers for 32-bits into the low bit number i.e., 1-bit, 2-bit, 4-bit, 8-bit, and 16-bit. In the area of deep learning, generally 32-bit float point number is the predominant numerical format used for research and deployment. However, the requirement of having low bandwidth and reducing the computing complexity of the quantization makes it necessity for the deep learning models that drive research into using lower-precision numerical formats.

**Figure 1:**
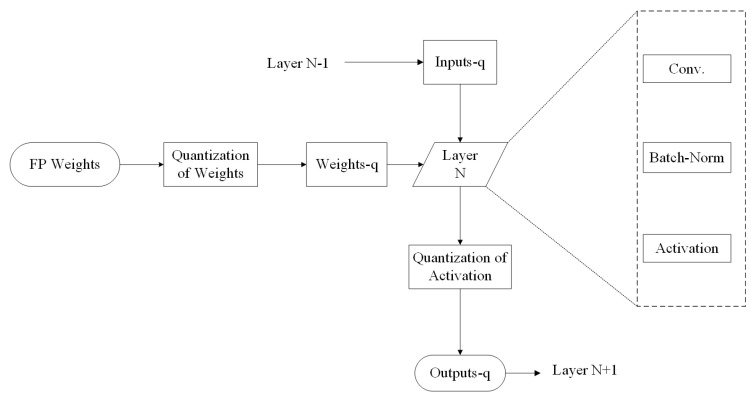
Schematic Diagram: Quantization Neural Network.

**Figure 2:**
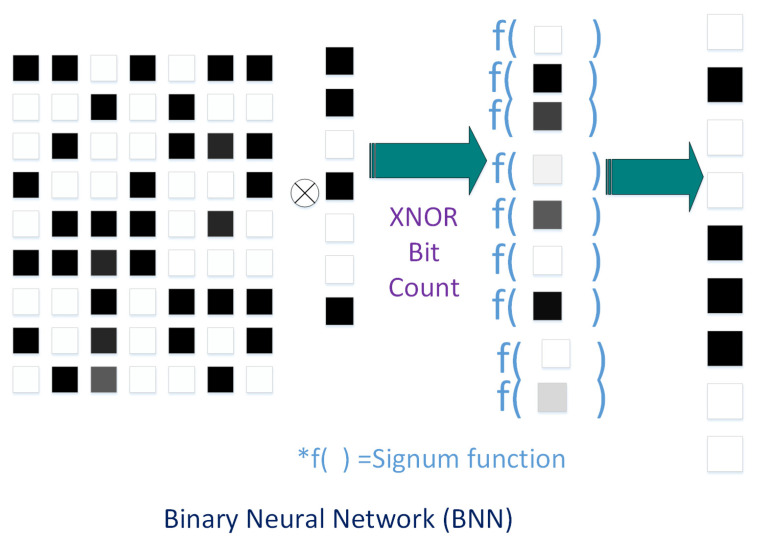
BNNs use binary weights and activations.

Figure 1 shows a quantization Neural Network block diagram. First, the full precision weights and activations are quantized. The total number of layers is “N+1”. The “layer *N*” is the content of the conv + batch-norm + activation. The operations in the *N* layer run with full precision within the boundaries. Courbariaux et al. [28] suggested 2-bit QNN, i.e., the binarization of 32 float points in 2-bit binary, which is called BNNs. In this methodology, both the weights and activations are binarized with 1-bit each. Since, only bit-wise operations are required, this reduces the need for memory storage and the computational complexity. As is shown in Figure 2, in the forward propagation, BNNs drastically reduce the memory size because most arithmetic operations can be compute with bit-wise operations, which means the power consumed is less than what is needed for full precision networks. In short, we can say that the BNNs provide power efficient networks.

### 2.1. Binarization Methods

The BNN, weights, and activations for each of the convolutional layers are quantized into 1-bit each, i.e., +1 or −1. Courbariaux et al. 2016 [28] have suggested two methods for the quantization of the full precision network into binary values. The first is the deterministic binarization method shown in Equation (1). The deterministic method is nothing but a simple signum function of weight and activations, where *x* is the floating-point (weights and activations) number, and $x^{b}$ is the binarized variable of *x*.

(1) $$x^{b}=Sign\left(x\right)=\{\begin{array}{cc} +1 & \mathrm{if}\;x\geq 0\\ -1 & \mathrm{others}. \end{array}$$

The second method of quantization of full precision as is shown in Equation (2), which is called stochastic binarization. This quantization method is more accurate and precise than the first one but it has more computational complexity.

(2) $$x^{b}=\{\begin{array}{cc} +1 & \mathrm{with}\;\mathrm{probability}p=\sigma (x)\\ -1 & \mathrm{with}\;\mathrm{probability}1-p, \end{array}$$

where σ is the hard-sigmoid function, as shown in the following Equation (3)

(3) $$\sigma \left(x\right)=clip\left(\frac{x+1}{2},0,1\right)=max\left(0,min\left(1,\frac{x+1}{2}\right)\right).$$

The deterministic method in Equation (1) is chosen in this research, because the stochastic binarization method requires hardware, that generates random bits during the quantization process, while the deterministic binarization method requires less computation and less memory space and has no need of a random bit generator during quantization.

### 2.2. Forward and Backward Propagation

**Figure 3:**
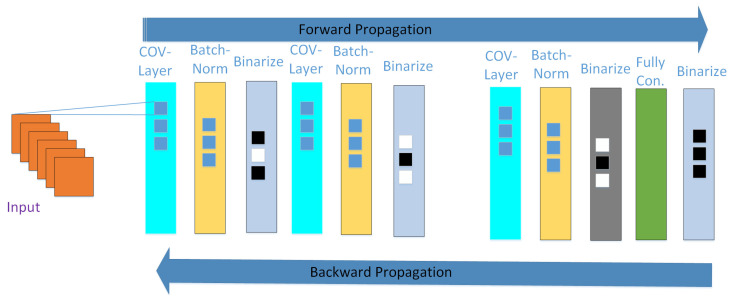
Binary Neural Network’s layout.

Figure 3 shows the BNNs architecture, which consists of different layers in the forward propagation of the first image input for the convolutional layer, while the output of convolutional layers is the input for the batch normalization layer, which becomes the output of the batch normalization to the binarization layers and finally to the fully connected layer. In this process, only the binary weights are used for the convolutional and fully connected layers. For the color image input, each channel for red, green, and blue is an 8-bit fixed-point value, for the first convolutional layer. Comparatively, the fixed continuous-valued input precision of *m* bits is simple to hold. For example, in the general case of *x* in an 8-bit fixed-point input and corresponding to that input $w^{b}$ is the binary weight, the output terms for that multiplication are given as Equation (4).

(4) $$s=x.w^{b},S=2^{n-1}\left(x^{n}.w^{n}\right),$$

where *x* is a vector of 320 × 280 8-bit inputs, $x_{1}^{8}$ is the first input’s most significant bit, $w^{b}$ is a vector of 320 × 280 1-bit weights, and s is the final weighted sum. Since the value of weight $w^{b}$ is +1 or −1 and input $x^{n}$ is binary-valued, the convolution is nothing but simple addition and subtraction. Thus, in the first convolutional layer, every neuron convolution is nothing but a part of chain of additions and subtractions. Furthermore, for all of the left convolutional layers, the input activations and weights are binarized. Hence, all multiply-accumulate operations are XNOR-addition operations, which is an essential ideal for FPGA LUT cell configuration. After completing the forward propagation process and loss evaluation, by running in backward propagation calculate all parameters associated with the gradient of the BNN. For the optimizing the deep learning methodology, the Stochastic Gradient Descent (SGD) process plays an essential role in the evaluation of real-valued gradients. SGD typically requires a lower learning rate. Equation (5), shows the relationship between the real-valued gradient and the updated weights.

(5) $$w^{t}=clip\left(w^{t-1}-\eta \frac{\partial c}{\partial w^{b}}\right),$$

where, $w^{t-1}$ = the old real-valued weight, η = the learning rate, $\frac{\partial c}{\partial w}$ = the gradient of cost concerning weight and $w^{t}$ is the updated weight.

Here, the generated weights are the crop to be in the range of −1 to +1. If the weight is falling out of the value between −1 and +1, it may fatten very much with each weight update. The large magnitude weights do not affect BNNs because the binarization of the weight always has to be in the range from −1 to +1.

One prominent drawback of the signum function is the zero derivative. The Straight-Through Estimator (STE) method has to overcome the zero gradient issues. To calculate the gradient STE applies a simple hard threshold function. Consider the signum function quantization, q=sign(r), and if $g_{\left(q\right)}$ is an estimator for the gradient $\frac{\partial C}{\partial q}$ has been obtained. Then, the straight-through estimator is simply $\frac{\partial C}{\partial r}$, which is given in Equation (6).

(6) $$g_{r}=\{\begin{array}{cc} +1 & \mathrm{if}-1\leq r\geq 1\\ 0 & \mathrm{others}. \end{array}$$

This stores only the gradient’s information, and for large values of r, it eliminates the gradient. STE performs back-propagation, and during this process, it assumes the derivative of the signum function is equal to one.

Since in this research, we use binarization for data pruning, it should be noted that other techniques exist for data pruning, such as Principal Component Analysis (PCA) [29] and Locally Linear Embedding (LLE) [30]. Moreover, we can also optimize the data size using the Restricted Boltzmann Machine (RBM) combined with the CNN [31,32]. All of the above-discussed pruning techniques help to reduce the data size. However, in our case, we use the binarization technique reduces computational complexity as well. The binary valued weights and activations require only addition and subtraction for the most of operations.

## 3. The BNN Related Models

In the section, we conduct a brief literature survey on the BNNs. To improve the BNNs accuracy and performance, several studies and research have been done. These studies are constructive and provide significant advantages in terms of strengthening the BNNs.

Rastegari et al. [33] proposed an XNOR-Net model similar to BNNs with the addition of a gain factor that improves the loss of information during the binarization process. Before the binarization of the weights and activations, this gain factor extracts them statically. Zhou et al. [34] suggested the DoReFa-Net model which has a variable quantization for the bit size. This method emphasizes the speed of the training time, unlike the XNOR-Net, where the gain factor is associated with only the weights. Hence it provides a more efficient inference without changing parameters (weights and gain). Tang et al. [35] suggested a methodology using binarized AlexNet with a high compression rate and better accuracy performance for large datasets like the ImageNet dataset. They suggested how to manipulate the three parameters of the learning rate, the scale factor, and better recognition to speed up training. Lin et al. [36] suggested a method using BNNs called the ABC-Net Model. They focused on restoring the accuracy difference between the BNNs and full precision networks. This model uses the combination of two ideas: one is multi-bit quantization and the second related to gain factors. This is combination model of DoReFa-Net and XNOR-Net. They claimed that the accuracy much close to the full precision network and had reduced parameter size. Darabi et al. [37] suggested an improved version of the classical BNN with some core principles advanced through the alternative of the State Through Estimator (STE). During the backpropagation, STE uses a unit function to evaluate the gradient through the signed activation layer. The problem concerning large activations is solved by clipping the activations beyond 1. They said that the accuracy is 86.5% and 91.3% for the CIFAR-10 and AlexNet datasets, respectively.

### A Comparison of BNN Methods

This subsection summarizes the accuracy of the different BNNs methodology on the CIFAR-10 and ImageNet datasets. The different levels of accuracy are associated with studies reported by different authors. Table 1 shows the accuracy of the CIFAR-10 database for the combination of topology and methodology. After the binarization of the full precision from Table 1, we can conclude that Courbaiaux et al. 2015 method achieved the most accuracy performance on the CIFAR-10, among all. The accuracy comparison for the ImageNet database is shown in Table 2 with the different methodologies associated with each particular topology. From Table 2, it’s clear that Courbaiaux et al. 2015 method has better Rank-1 accuracy and performance over all other methods for the AlexNet topology. Hence, for this research, Courbaiaux et al. 2015 methodology is used.

## 4. The Proposed Sub-Optimal Method for Region Segmentation

In this section, we first, discuss Binary Fully Convolutional Neural Networks and review its architecture. We then the introduce of the Taguchi method and the best possible quantization combination for one or more convolution layers. We also outline our methodology, i.e., Binary Fully Convolutional Neural Networks for the region segmentation for robotica vision at UGV. Last, we describe the model PYNQ-FPGA accelerator for UGV region segmentation.

### 4.1. Binary Fully Convolutional Neural Networks (B-FCN)

**Figure 4:**
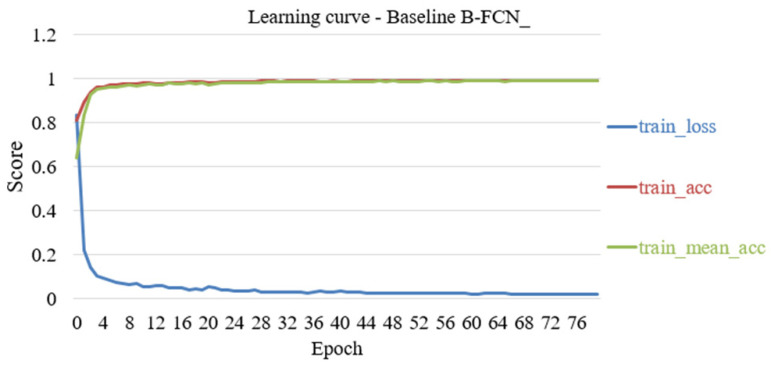
Baseline B-FCN iterative 80 times training results.

**Figure 5:**
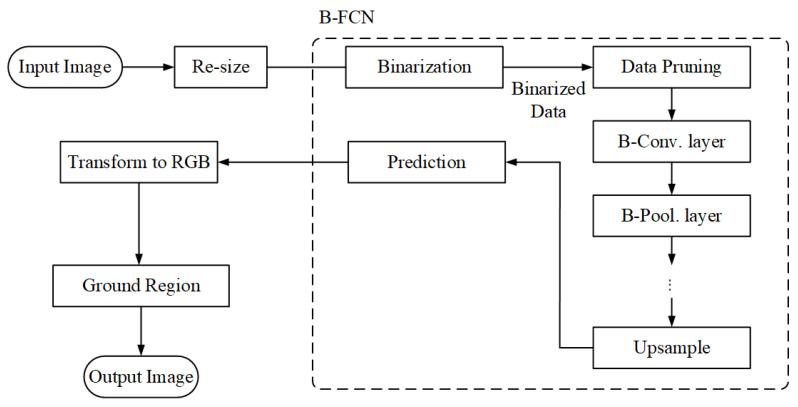
Flow Architecture of B-FCN to ZCU104 SoC FPGA.

A Binary Fully Convolutional Neural Network (B-FCN) is a Fully Convolutional Neural Network. It is a low precision FCN with 2-bit (binary). First, we focused on Fully Convolutional Neural Network (FCN) and then employed quantization on the FCN to get a B-FCN module. In terms of the actual training results, the recognition accuracy of FCN-8s is the highest. Figure 4 shows the baseline B-FCN training results of 80 times iterations. As it is shown in Figure 5, the binary neural network architecture can be used to integrate the Fully Convolutional Neural Networks computing model (B-FCN) to reduce the execution time of the Xilinx ZCU104 AI SoC-FPGA. The method not only dramatically reduces the computing time, but the computing results also are similar to the Fully Convolutional Neural Networks architecture, which causes a computational decrease and an improvement in computing performance.

### 4.2. The Taguchi Method Based Binary Fully Neural Networks (TM-B-FCNs)

In this subsection, we first, discuss the Taguchi method. Using the Taguchi technique, we find the required parameters for the set or sets of binary convolutional case or cases. The Taguchi method is an analytical approach for a design algorithm first proposed to enhance the quality of products in manufacturing. Over the last few years, it has also been applied to various areas of design to improve any kind of design process or system in engineering, science, and marketing. The quantization of CNNs, i.e., BNNs essentially involves finding the required kernel size and the pruning size for optimizing the accuracy, storage, power-efficiency, and computational complexity. The Taguchi method is an approach for finding relevant factors like the bit length and kernel size for the optimal conditions.

The quantization of CNNs, i.e., BNNs essentially involves finding the required kernel size and pruning size for optimizing the accuracy, storage, power efficiency and computational complexity. Here the proposed Taguchi based B-FCN take up two factors: one is storage size and the second is the accuracy of the B-FCN system. As is shown in Table 3 there are a total of 18 (say L1–L18) optimal designs for the experiments. For this study, the table shows there are two convolutional layers, B-Conv1 with three kernels sized 13 × 13, 11 × 11, and 9 × 9 size and B-Conv2 with three kernels sized 7 × 7, 5 × 5, and 3 × 3 at two levels of different data pruning accuracy and storage. In terms of accuracy, L1 is the best case and L18 is the worst case. First, we set the maximum accuracy (L1) and then we looked for when the accuracy reached 80%, i.e., the minimum accuracy (L15) condition for our requirements. Now, we compare this with the maximum accuracy, L1, with baseline L4, and the accuracy decreases only by 2.78% and the storage memory required decreases by 2.03%. Similarly comparing L4 and the minimum accuracy from the table, the accuracy of baseline L4 increases by 2.50% with 4.04% extra storage memory. Thus, we find that L4 is the best case for our requirements.

## 5. Experimental Results

For comparison, in total, we evaluated 6080 indoor testing scenes for UGV robot vision testing were evaluated based on three types of data sets KITTI 580 images, CamVid 3500 images, and MIT 2000 images. The complex environments included indoor like patterned floors, shadows, and reflections, and outdoor like obstacle detection module, path planning, and tracking module. These scenes were all detectable. Overall, the UGV robots benefited from better navigation and route planning when using our approach. All image scenes were annotated for the floor and non-floor regions on the ground. For evaluating the results of the proposed methodology, we trained 5500 indoor testing sets in BNN+ and BNN. Moreover, 580 sets were used to evaluate the accuracy. Here, true positives, false positives, true negatives, and false negatives were calculated. Besides, we evaluated these using the well-known accuracy in Equation (7), and looked for the additional G-Mean reliability of the accuracy was also evaluated, as it is shown in Equation (8). Table 4 shows the performance results for the traditional Fully Convolutional Neural Network with the proposed B-FCN in Accuracy and G-Mean.

(7) $$Accuracy=\frac{TruePositive+TrueNegative}{TotalPopulation}$$

(8) $$\begin{array}{cc} Sensitivity & =\frac{TruePositive}{Truepositive+FalseNegative},\\ Specificity & =\frac{TrueNegative}{FalsePossitive+TrueNegative},\\ G-Mean & =\sqrt{SensitivityxSpecificity} \end{array}$$

**Figure 6:**
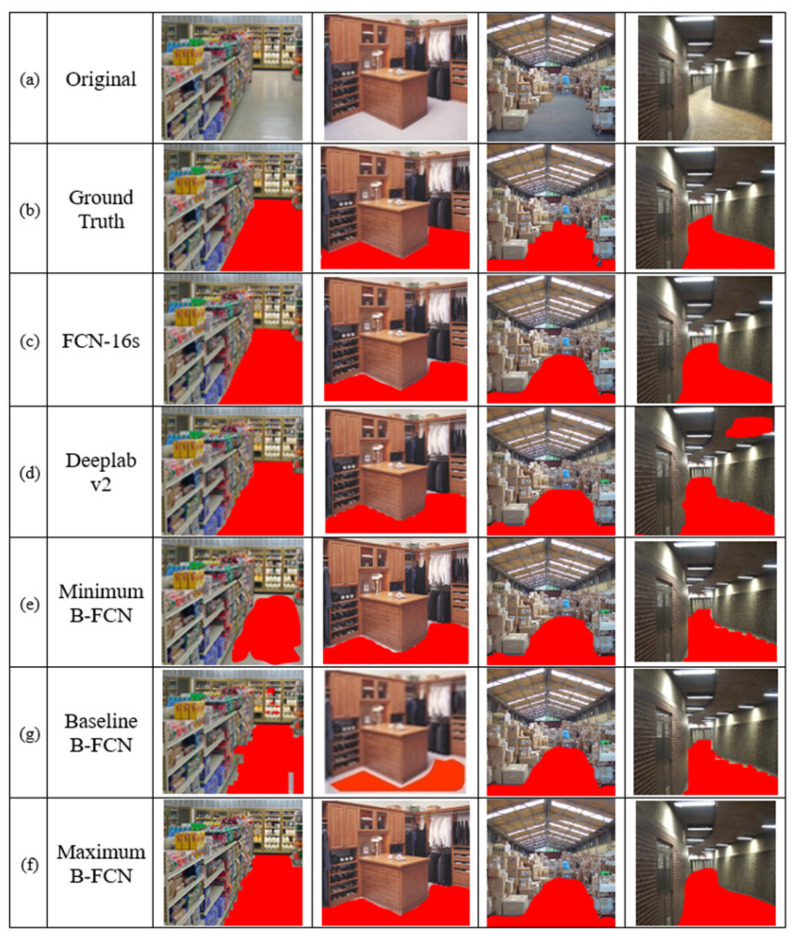
Compare the different floor segmentation method results.

As is shown in Figure 6, the results are shown in terms of comparing the different methods the Baseline B-FCN with the Maximum B-FCN and the Minimum B-FCN. The walkable floor area was identified with the error. Therefore, the area edge detection algorithm was used to flatten the edges of the detection floor area. Finally, an improved Taguchi based B-FCN was combined to take advantage of various detection methods to obtain the optimal floor area position. The proposed method exerted its effect on the detection of the floor area position detection and effectively improved the accuracy of identification.

The compression of the binary platform prototype design with the different datasets is shown in Table 5. This table presents a synthesis of the different binary platform results regarding of accuracy, storage size, and power consumption. In this paper, we synthesized three types of B-FCN i.e., the Minimum B-FCN, the Baseline B-FCN and the Maximum B-FCN. First, we set up maximum and the minimum accuracy. The minimum accuracy should be higher than 80%. Using the Taguchi method we searched for the Baseline B-FCN, which was the most balanced sub-optimal condition that improved storage size with significantly less of a loss of accuracy within the range of 1–3%. We can see from the table the number of BRAM for the Baseline B-FCN decreased by 32% and accuracy compensation was 2.78%, when compared to the Maximum B-FCN. The proposed method for B-FCN has a higher GOPS/W compared with all other architectures, which means it is a more power-efficient architecture for PYNQ ZCU104 FPGA in terms of the real-time embedded system. In this way, the experimental results prove that the proposed B-FCN is a much more power-efficient architecture at this level of accuracy.

### 5.1. System Architecture

**Figure 7:**
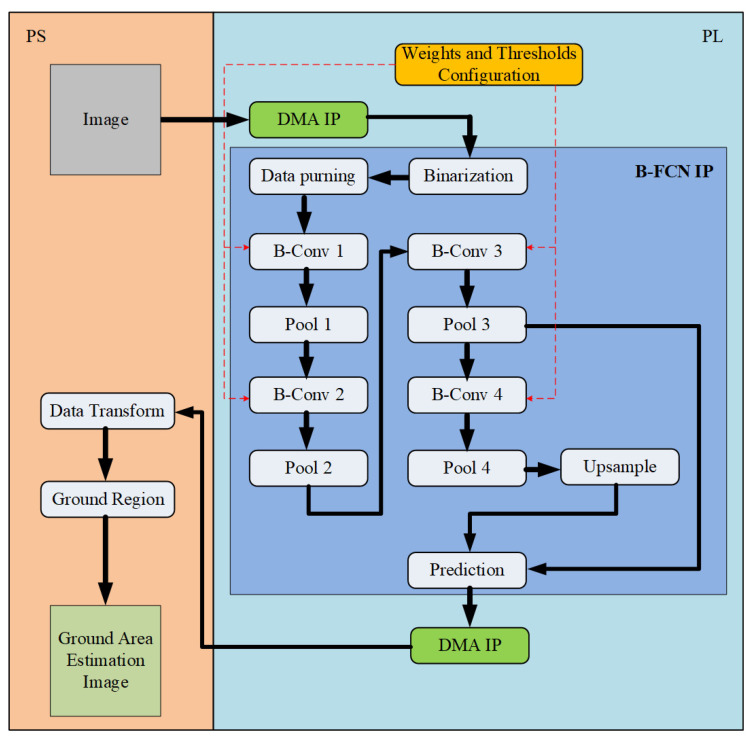
The proposed B-FCN system architecture on PYNQ ZCU104 FPGA.

Figure 7 shows the B-FCN system architecture on the PYNQ ZCU104 FPGA [38]. It belongs to the research and development platform in the UltraScale + series, and the main multi-core processor system on chip (Multiprocessor System on Chip—MPSoC) is used for the base architecture. It is also made by TSMC 16FinFET to realize the Zynq UltraScale + MPSoC, which is used in Zynq-7000, and based on the SoC series. The system began with the initialization of the system environment, import the underlying bitstream file and various function modules. Then, it imported the video or image into the B-FCN computing core, and configured the B-FCN endpoint starting the calculation, and finally, the calculation result was founded and displayed on the HDMI screen.

### 5.2. Hardware Implementation

First, the loads for the pre-placed bit file and the TCL file were placed into the PL side to configure the hardware architecture. Then, the PS side started to initialize the USB video camera, the underlying HDMI-related settings, and the B-FCN weight and threshold load. After the camera and the image were screened, the accessible ground area was judged through the AXI 4-Stream to the B-FCN. After the judgement was completed, the resulting image was sent to the PS terminal and put on the display screen.

**Figure 8:**
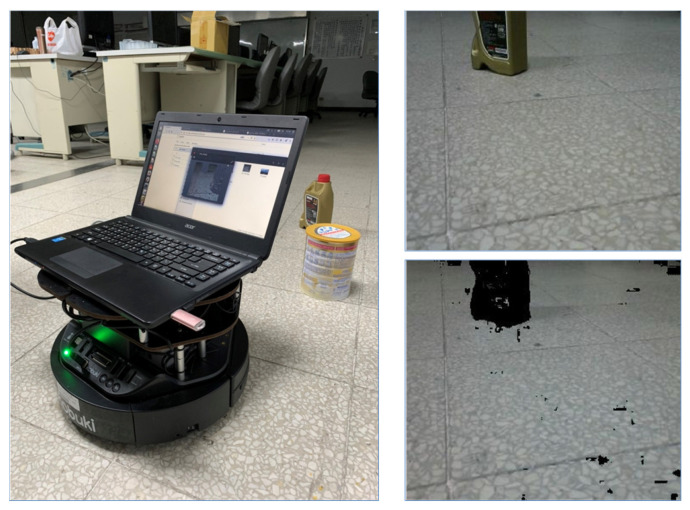
Proposed algorithm deploying to a ROS compatible TurtleBot-II UGV rover-style robot.

**Figure 9:**
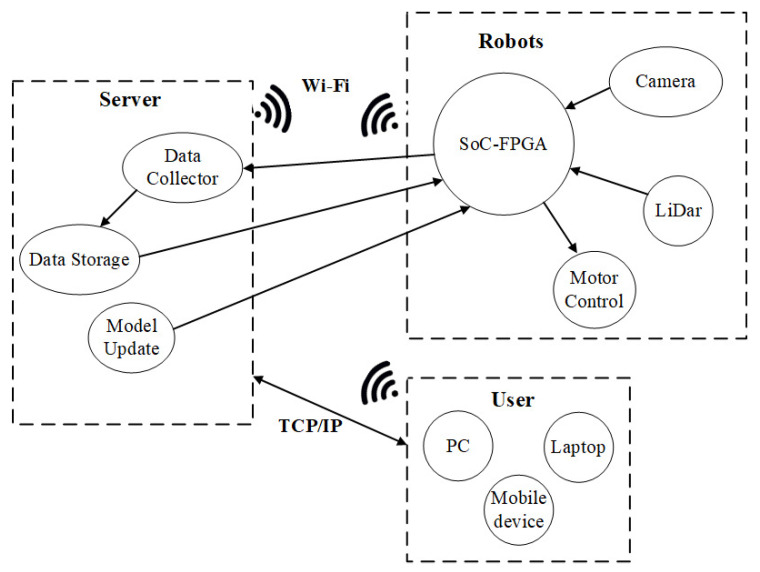
Distributed computation system for deploying real-time floor segmentation.

The Taguchi based B-FCN system was used for the unmanned vehicle as it is shown in Figure 8. The unmanned vehicle could be controlled through a computer connected to a wireless network, and it can also be controlled through a pre-loaded program. The main test was on the ground to see if it could travel the region and identify anything in its way. Communication was based on Robot Operation System (ROS) and communications infrastructure nodes communicated with each mode node. A schematic diagram of the hardware system shown in Figure 9. While running the unmanned vehicle the robot recognized the image of the floor, and the non-travel area was marked as the black region and displayed on the screen.

As it is shown in Table 5, we can compare the different platforms with CNN and binary CNN architectures used for image classification. We know that the FCN architecture was 2 to 3 times more complex architecture than CNN. From the table, we can see that the binary CNN retained an accuracy of up to 66.80%, while using of 2210 BRAM with 74.96 GOPS/W for the ImageNet dataset for classification. However, the proposed B-FCN architecture retained an accuracy of up to 87.58% with 451.79 GOPS/W while only using 213 BRAM for full segmentation. In other words, the B-FCN architecture retained a high level of accuracy using only 9.6% of BRAM and was 6 times more power efficient compared to binary CNN. In terms of memory storage and power consumption, the proposed B-FCN architecture proved that it was efficient for KITTI, MIT, and CamVid datasets, because it needed minimal storage and very little power to compute its operations.

The proposed architecture provided about 85% average accuracy for region segmentation over UGV, which is an acceptable level of accuracy for the robot system. One of the most beneficial advantages of our B-FCN architecture is its low level of power consumption. The large size of the data should have required a high resolution, resulting in higher throughput. This typically placed a more significant burden on the battery of the robot. In other words, this made for power-hungry robots. Thus, the proposed architecture reduces the data’s size resulting in lower power uses by the robot for region segmentation. The disadvantage of the UGV was that it left the boundary while moving, but its moving system was not highly affected by the segmentation.

We discussed in Section 2 the pruning techniques like PCA, LLE and, RBM. In Table 3, we can easily see that only more or less of 2% of LUTs and 18% FF were required for our proposed B-FCN architecture after binarization pruning. Therefore, there was no need for node pruning. When we applied node pruning, this helped reduce the size but caused a more significant amount of design complexity. Hence, there was no further node pruning that was needed.

## 6. Conclusions

In this paper, a region segmentation algorithm is proposed. The BNNs provide an essential model of improving storage and making faster inferences than conventional DNNs. The BNN’s accuracy is not as great as the full precision models, and our refinements have overcome the issues that have arisen. The FPGA is reconfigurable and has a parallel architecture, which allows the neural network architecture to have great scalability and adaptability. The proposed algorithm accuracy attains upwards of 87.58% B-FCN and Taguchi B-FCN 84.80%, for the SoCFPGA embedded platform, and it can reach 25 FPS with 1080P resolution. Regarding the experimental results, the storage size was much improved, with up to 32% decrease compared to the Maximum B-FCN. The second most significant part of this proposed sub-optimal methodology is power consumption. This proposed methodology is more better power efficiency i.e, it has 451.79 GOPS/W compared to other binary platforms with a higher throughput of 2135 GOPS.

After the actual image is imported by the video camera, the B-FCN calculates the feasible range of the ground. The FPGA hardware IP setting is done, and simple optimizations are performed to calculate the SoC-FPGA ground feasibility area identification. Comparing the different cases, we understand the application feasibility of the B-FCN proposed in this paper for the embedded platform, which is essential for future applications. The application of SoC-FPGA has contributed to new solutions, even though most of the practical cases of object recognition provided in the SoC-FPGA field have belonged to a small group. Most of the previous studies used the CPU or GPU for ground classification and recognition, and the calculation accuracy of the CPU and GPU are higher than the calculation results published in this research. However, the resource usage and power consumption required for these calculations are relatively high. Compared with the resource usage and power consumption in the FPGA, it is low for system stability. The FPGA is also better because these characteristics will lead to more in-depth research and discussion in the field of SoC-FPGA.

In future work, we will make the B-FCN more efficient by updating the binary and quarternary FCN (B-Q-FCN) and removing less significant nodes. Moreover, we can use a pruning method other than binarization, such as RBM and PCA for larger datasets, which will maximize the complexity of architecture. For implementation, we can use the next-generation FPGA, which will be speed up the system.

## Figures and Tables

**Table 1:** Accuracy of different methodology on the CIFAR-10 database.

Ref.	Methodology	Topology	Accuracy (%)
[11]	Simonyan et al. Full Precision	FP32	97.09
[13]	Courbaiaux et al. BNN	BNN	89.85
[33]	Rastegri et al. XNOR-Net	BNN	89.83
[34]	Zhou et al. BNN+	DoReFa-Net	87.16
[37]	Darabi et al. BNN+	AlexNet	87.16

**Table 2:** Accuracy of different Methodology on the ImageNet database.

Ref.	Methodology	Topology	Rank-1 Acc. (%)	Rank-5 Acc. (%)
[10,11]	Krizhevsky et. al. and Simonyan et. al. Full Precision	GoogleNet	71.3	90.0
		AlexNet	57.1	80.2
		ResNet18	69.3	89.2
		ResNet34	73.3	91.3
		ResNet50	76.1	92.8
[13]	Courbaiaux et al. BNN	GoogleNet	47.1	69.1
		AlexNet	47.8	67.1
[33]	Rastegri et al. XNOR-Net	ResNet18	51.2	73.2
		AlexNet	44.2	69.2
[34]	Zhou et al. DoReFa-Net	AlexNet	43.6	-
[37]	Darabi et al. BNN+	AlexNet	46.11	75.70
		ResNet18	52.64	72.98

**Table 3:** Taguchi based forecast matrix of design of experiment.

S.No.	B-Conv1	B-Conv2	Data Pruning	Accuracy (%) Tensorflow	Prediction Hardware Size (%)			Prediction
					LUTs	FF	DSP	
***1***	**13 × 13**	**7 × 7**	**96 × 96**	***87.58***	***2.38***	***20.23***	***0.93***	***Maximum***
2	13 × 13	5 × 5	96 × 96	86.21	2.15	18.20	0.87	
3	13 × 13	3 × 3	96 × 96	86.23	1.92	16.18	0.81	
***4***	**11 × 11**	**7 × 7**	**96 × 96**	***84.80***	***2.15***	***18.20***	***0.87***	***Baseline***
5	11 × 11	5 × 5	96 × 96	84.58	1.92	16.18	0.81	
6	11 × 11	3 × 3	96 × 96	84.24	1.69	14.16	0.75	
7	9 × 9	7 × 7	96 × 96	81.33	1.92	16.18	0.81	
8	9 × 9	5 × 5	96 × 96	81.29	1.69	14.16	0.75	
9	9 × 9	3 × 3	96 × 96	80.82	1.46	12.14	0.69	
10	13 × 13	7 × 7	48 × 48	85.27	2.35	20.23	0.75	
11	13 × 13	5 × 5	48 × 48	85.19	2.12	18.20	0.69	
12	13 × 13	3 × 3	48 × 48	84.32	1.89	16.18	0.64	
13	11 × 11	7 × 7	48 × 48	84.79	2.12	18.20	0.69	
14	11 × 11	5 × 5	48 × 48	84.28	1.89	16.18	0.64	
***15***	**11 × 11**	**3 × 3**	**48 × 48**	***82.30***	***1.65***	***14.16***	***0.58***	***Minimum***
16	9 × 9	7 × 7	48 × 48	79.13	1.89	16.18	0.64	
17	9 × 9	5 × 5	48 × 48	79.81	1.65	14.16	0.58	
18	9 × 9	3 × 3	48 × 48	78.11	1.42	12.14	0.52	

**Table 4:** Different methods of floor segmentation result point using on UGV.

Method	Bit Length	Accuracy (%)	G-Mean (%)
FCN-8s	32	97.58	96.86
DeepLabv2	32	97.13	95.80
Minimum B-FCN	2	82.30	81.9
Baseline B-FCN	2	84.80	83.7
Maximum B-FCN	2	87.58	86.6

**Table 5:** Comparison of the design prototype of planing proposal with binary platform using different datasets.

Architecture	Platform	Device	Dataset	Bit	Acc. (%)	k-LUTs	BRAM	GOPS	GOPS/W
AlexNet-CNN	CPU	I7-6700	SVHN	32	97.10	-	-	69	0.76
			CIFAR-10		88.58				
AlexNet-CNN	GPU	Nvidia GTX1070	SVHN	32	97.10	-	-	2708	9.25
			CIFAR-10		88.58			3380	24.7
FINN-CNN	FPGA	Xilinx ZC706	SVHN	1	94.90	46.2	186	2465	684.7
			CIFAR-10		80.00				
FBNA	FPGA	Xilinx ZC702	SVHN	2	96.90	29.6	103	2236	699
			CIFAR-10	1	88.61			722	219
QNN	GPU	Nvidia GTX750	ImageNet	2	77.76	-	-	-	-
FP-BNN	CPU	Xeon E5-2640	MINST	8	98.15	-	-	95	18.80
			CIFAR-10		89.13	-			191.29
			ImageNet		79.04				168.35
	FPGA	Alttrea Statix V	MINST	8	98.24	68.35	2210	26.2	225.36
			CIFAR-10		86.31				358.64
			ImageNet		66.80				74.96
Minimum B-FCN	FPGA	Xilinx ZCU104	KITTI, MIT, CamVid	2	82.30	107.2	112	1002	302.71
Baseline B-FCN					84.80	111.7	144	1142	330.05
Maximum B-FCN					87.58	144.3	213	2135	451.79
